# Identification of the zebrafish homologues of IMPG2, a retinal proteoglycan

**DOI:** 10.1007/s00441-023-03808-z

**Published:** 2023-07-20

**Authors:** M. E. Castellini, G. Spagnolli, L. Poggi, E. Biasini, S. Casarosa, A. Messina

**Affiliations:** 1 Department of Cellular, Computational and Integrative Biology (CIBIO), University of Trento, Via Sommarive, 9, 38123 Povo, TN Italy; 2 Sibylla Biotech S.R.L, Piazzetta Chiavica 2 - 37121, Verona, VR Italy; 3 Centre for Medical Sciences (CISMed), University of Trento, Via S. Maria Maddalena, 1, 38122 Trento, TN Italy; 4 Centre for Mind/Brain Sciences (CIMeC), University of Trento, Piazza Manifattura 1, 38068 Rovereto, TN Italy

**Keywords:** Zebrafish retinal development, Photoreceptors, Interphotoreceptor matrix proteoglycan 2, Inherited retinal dystrophies, Homology modelling

## Abstract

Photoreceptor outer segments are surrounded by a carbohydrate-rich matrix, the interphotoreceptor matrix, necessary for physiological retinal function. Few roles for molecules characterizing the interphotoreceptor matrix have been clearly defined. Recent studies have found the presence of nonsense mutations in the interphotoreceptor matrix proteoglycan 2 (*IMPG2)* gene in patients affected by retinal dystrophies. *IMPG2* encodes for a proteoglycan synthesized by photoreceptors and secreted in the interphotoreceptor matrix. Little is known about the structure and function of this protein, we thus decided to characterize zebrafish *impg2*. In zebrafish there are two Impg2 proteins, Impg2a and Impg2b. We generated a phylogenetic tree based on IMPG2 protein sequence similarity among vertebrates, showing a significant similarity between humans and teleosts. The human and zebrafish proteins share conserved domains, as also shown by homology models. Expression analyses of *impg2a* and *impg2b* show a continued expression in the photoreceptor layer starting from developmental stages and continuing through adulthood. Between 1 and 6 months post-fertilization, there is a significant shift of Impg2 expression toward the outer segment region, suggesting an increase in secretion. This raises intriguing hypotheses about its possible role(s) during retinal maturation, laying the groundwork for the generation of most needed models for the study of IMPG2-related inherited retinal dystrophies.

## Introduction

In every tissue, extracellular matrix components interact with cells to finely control and regulate cell and tissue functions. In the retina, the extracellular matrix surrounding photoreceptors' outer segments (defined as IPM, InterPhotoreceptor Matrix) is highly specialized. It is mainly composed of proteoglycans and glycosaminoglycans, synthesized by the surrounding photoreceptor cells, retinal pigment epithelial (RPE) cells and Müller glia cells (Mieziewska 1996), and its involvement in retinal function and diseases has only recently started to be understood (Al-Ubaidi et al. 2013; Ishikawa et al. 2015). The normal function and metabolism of photoreceptor cells depend on several different interactions with the RPE, including exchange of metabolites and catabolic by-products, retinoid transport, alignment and adhesion of photoreceptor outer segments (OS) to the RPE, and regulation of OS disk shedding (Hollyfield et al. 1990; Uehara et al. 1990). Many of these processes follow the environmental light–dark transition and rely on IPM components. Studies performed by Uehara et al. (1990) showed light-evoked changes in the distribution of glycoproteins and proteoglycans in the IPM, which shift away from the outer segment toward the RPE and inner segments upon light stimulation, to guarantee a better transport of substances such as retinoids from the photoreceptor outer segments to the RPE. The IPM was also found to play key roles in intercellular communication, regulation of neovascularization, and photoreceptor maintenance and survival (Hewit and Adler 1989; Inatani and Tanihara 2002; Inoue et al. 2006; van Huet et al. 2014; Felemban et al. 2018). Mutations in proteins localized to the IPM such as interphotoreceptor retinoid-binding protein, IRBP, are involved in inherited retinal dystrophies (IRDs) (den Hollander et al. 2009; Li et al. 2013; Sato et al. 2013; Markand et al. 2016). Indeed, IRBP plays a crucial role in mediating extracellular diffusion of retinoids during the retinoid cycle (Gonzalez-Fernandez 2003; Zeng et al. 2020) and it participates in lipid transport across the IPM (Ghosh et al. 2015; Zeng et al. 2020).

Recent studies have reported that mutations in the human interphotoreceptor matrix proteoglycan (*IMPG2)* gene are associated with autosomal recessive Retinitis pigmentosa (arRP) (Bandah-Rozenfeld et al. 2010; van Huet et al. 2014) and autosomal dominant and recessive Vitelliform macular dystrophy (VMD) (Meunier et al. 2014; Brandl et al. 2017). RP [MIM 268000] is the most common inherited retinal dystrophy (IRD) (Bundey and Crews 1984; Haim 2002; Hartong et al. 2006) involving progressive degeneration of photoreceptor cells and retinal pigment epithelial (RPE) cells (Ferrari et al. 2011; Daiger et al. 2013). VMD [MIM 153700], also called Best disease, is an early-onset disorder characterized by accumulation of lipofuscin-like material within and beneath the RPE together with a progressive loss of central vision (Sun et al. 2002; Querques and Souied 2016). The *IMPG2* gene encodes for the proteoglycan IMPG2, synthesized by rods and cones and secreted and/or exposed by these cells in the IPM (Hollyfield 1999; Foletta et al. 2001; Chen et al. 2003, 2004). Biochemical studies suggest that the IMPG2 proteoglycan directly binds to hyaluronan and chondroitin sulphate. Analysis of IMPG2 mRNA and protein revealed that it is expressed exclusively by photoreceptor cells in the retina and pinealocytes in the pineal gland. IMPG2 might play a role in maintaining the structural integrity and organization of the IPM and may promote the growth and maintenance of the light-sensitive photoreceptor outer segment (Foletta et al. 2001). In more recent studies, IMPG2 null mice display progressive cone degeneration, increased levels of endoplasmic reticulum (ER) stress-related proteins, and abnormal accumulation of the interphotoreceptor proteoglycan 1 (IMPG1) at the subretinal space, leading to reduced visual function (Salido and Ramamurthy 2020; Xu et al. 2020). However, the role of IMPG2 in retinal development and function remains to be better elucidated.

In this study, we analyzed Impg2 expression and protein structure in the zebrafish retina. Zebrafish has a cone-enriched retina as well as a cone-dominant vision as its human counterpart, although it lacks a cone-only central structure comparable to the human fovea. This makes the zebrafish a very suitable organism to model retinal degenerative diseases affecting survival, integrity and function of photoreceptors in humans (Fadool and Dowling 2008; Avanesov and Malicki 2010; Gestri et al. 2012; Chhetri et al. 2014; Noel et al. 2021).

Zebrafish has two Impg2 paralogues, Impg2a and Impg2b, due to the major event of whole-genome duplication that occurred in most teleosts (Glasauer and Neuhauss 2014). We thus performed a phylogenetic analysis of related IMPG2 proteins from different vertebrate species to investigate the extent of protein conservation during evolution. Moreover, since IMPG2 protein structure has largely been unstudied, we also performed homology modelling of IMPG2 conserved domains in humans and zebrafish. Finally, we analyzed the expression of *impg2a* and *impg2b* mRNAs and proteins during early development and in adulthood.

## Materials & methods

### Animal care and maintenance

AB/TU wild-type zebrafish strain was used for all experimental procedures. Zebrafish were used under the approval of the Animal Welfare Body (OPBA, Organismo Per il Benessere Animale) of the University of Trento and Ministero della Salute (Project Number 151/2019-PR) and were raised following standard procedures (Westerfield 2000).

### Phylogenetic tree

NCBI database was used to find orthologs to the human IMPG2 (human IMPG2, NP_057331.2; rat IMPG2, XP_008766850.1; mouse IMPG2, XP_017172459.1; chicken IMPG2, XP_015151604.1; western clawed frog IMPG2, XP_012813076.1; channel catfish IMPG2, XP_017325640.1; channel catfish IMPG2-like, XP_017313887.1; Japanese medaka Impg2a, XP_023806900.1; Japanese medaka Impg2b, XP_023805177.1; Asian swamp eel IMPG2, XP_020464932.1; Asian swamp eel IMPG2-like, XP_020447553.1; barramundi IMPG2, XP_018558135.1; barramundi IMPG2-like, XP_018517497.1; spotted gar IMPG2, XP_015219513.1; zebrafish Impg2a XP_017213311.1; zebrafish Impg2b XP_021329195.1; XP_015821571.1; blind cave fish IMPG2, XP_022521341.1; turquoise killifish IMPG2, XP_015821571.1). After choosing the vertebrate species to be included in the phylogenetic tree, IMPG2 protein sequences of these animals were obtained from NCBI (https://www.ncbi.nlm.nih.gov/) and a multiple protein sequence alignment was performed by using Clustal Omega sequence alignment program, provided by EMBL-EBI (https://www.ebi.ac.uk/Tools/msa/clustalo/). The same sequence analysis tool was used to generate a phylogenetic tree, based on protein sequence similarity.

### Modelling of SEA and EGF-like domains

Modelling of the SEA (Sperm protein, Enterokinase and Agrin) and EGF (epidermal growth factor)-like domains was performed by using the iTasser webserver (https://zhanglab.ccmb.med.umich.edu/I-TASSER/) (Yang and Zhang 2015). The submitted sequences for human IMPG2 were: 239–390 (SEA1), 896–1012 (SEA2) and 1012–1098 (EGF-like tandem repeat). The corresponding submitted sequences of Impg2a were 239–371 (SEA1), 775–889 (SEA2) and 889–972 (EGF-like tandem repeat). The corresponding submitted sequences of Impg2b were 1177–1333 (SEA1), 2473–2587 (SEA2) and 2587–2673 (EGF-like tandem repeat).

### RNA extraction and RT-qPCR

Total RNAs from pools of 15 embryos at different developmental stages and pools of adult organs (pools of 3 eyes, 2 brains, 2 hearts, 1 digestive tract, 2 livers, 1 female and 1 male gonad, 2 swim bladders, 2 kidneys) were extracted by Macherey Nagel NucleoSpin^®^ RNA. cDNA was synthesized by Super-Script^®^ VILO™ cDNA Synthesis Kit (Invitrogen). RT-qPCR was performed using KAPA SYBR^®^ FAST Master Mix (KAPA Biosystems) according to the manufacturer’s instructions. *Ube2a* was used as housekeeping gene (Xu et al. 2016) and *rhodopsin* was used as reference gene since it is highly expressed in the retina. Relative expression of each mRNA with respect to *Ube2a* mRNA was calculated as the difference in cycle number between the gene of interest and the housekeeping gene, and as the average of three independent experiments. Expression analysis was performed using the CFX3Gene Manager (BioRad) software. Gene primers are listed in Table 1.

**Table 1:** Primers used for RT-qPCR experiments

**Gene name**	**Forward**	**Reverse**
*impg2a*	5’-GTTTGTGAAAAAGCTGGAGAC-3’	5’-CATCCAGGTCTGGTACTCTTCC-3’
*impg2b*	5’-TGAACCTTTTGCTGTATCATGG-3’	5’-CGGTTACAGGCACTACAATGTC-3’
*rhodopsin*	5’-AGCCCATACGAATACCCACA-3’	5’-CTTCTTGTGCTCGATGGTGA -3’
*Ube2a*	5’-TGACTGTTGACCCACCTTACAG-3’	5’-CAAATAAAAGCAAGTAACCCC-3’

### Protein extraction and western blot

Total proteins from pools of 15 embryos at different developmental stages and pools of 3 adult eyes and 2 adult brains were extracted using RIPA buffer. 10 µg of total extract were resolved by SDS-PAGE, transferred to a nitrocellulose membrane and then incubated with the antibodies reported in Table 2 according to the following protocol (Bosco et al. 2018).

**Table 2:** Antibodies used for Western blot (WB) and immunohistochemistry (IHC) experiments. RRIDs were obtained at https://scicrunch.org/resources

**Antibody**	**Use**	**Dilution**	**Producer/cat. #**	**Producer/cat. #**
Rabbit polyclonal anti-hIMPG2	IHC, WB	1:200 (IHC), 1:1000 (WB)	AbCam, AB82813	AB_1860700
Mouse monoclonal anti-Rhodopsin 1D4	WB	1:1000 (WB)	Sigma, R5403	AB_477464
Mouse monoclonal anti-Zpr1	IHC	1:500	ZIRC, zpr-1	AB_10013803
Mouse monoclonal anti-Zpr3	IHC	1:500	ZIRC, zpr-3	AB_10013805
Mouse monoclonal anti-α actinin (H-2)	WB	1:8000	Santa Cruz, sc-17829	AB_626633
Goat anti-rabbit Alexa 594	IHC	1:1000	Life Technologies, A11034	AB_2576217
Goat anti-mouse Alexa 488	IHC	1:1000	Life Technologies, A11037	AB_2534095
Goat anti-rabbit IgG (H+L) Biotin-SP	WB	1:500	Jackson ImmunoLab, 111-035-144	AB_2307391

### Immunohistochemistry

Embryos at different stages and eyes dissected from adult fishes at different stages were fixed in 4% paraformaldehyde (PFA) at 4 °C overnight, embedded in 30% sucrose at 4 °C for 2–3 h and included in OCT compound. A Leica cryostat was used to obtain 14 µm retina sections. Immunohistochemistry was performed as follows: slides with sections were incubated in blocking solution (0.1% Triton X-100 and 0.5% BSA in 1 × PBS) for 1 h at room temperature (RT) and then incubated in diluted primary antibody in blocking solution (dilution specific for the primary antibody in use), at 4 °C overnight in a humidified chamber. After 3 washes of 10 min in 1 × PBS, 0.1% Triton X-100, slides were incubated in secondary antibody in blocking solution (1:1000), for 2 h at RT in a humidified chamber. Slides were then washed 3 times for 10 min 1 × PBS, 0.1% Triton X-100 and then incubated with nuclear staining dye (1:10,000; Hoechst 1) in 1 × PBS for 10 min at RT. Following 3 washes of 10 min in 1 × PBS, 0.1% Triton X-100, slides were mounted using Aqua-Poly/Mount coverslipping medium (Polysciences, Inc.). As negative controls, sections were incubated with secondary antibodies only and revealed no detectable signal (not shown).

Primary and secondary antibodies used for immunohistochemistry experiments are reported in Table 2.

### Image acquisition and analyses

Measurements of the length of Impg2 signal were done on images of IHC experiments for Impg2 and Zpr-3. Three eyes dissected from three different animals were used, and three sections were considered for each eye. For each section, three different regions of the retina (two peripheral, one central) were considered to have a total number of measurements equal to 27. Measures included: i) the distance between the outer limiting membrane (OLM) and the more external Impg2 signal; ii) the distance between the (OLM) and the outer limit of the rod outer segments (ROS), identified by the outer limit of Zpr-3 staining.

Immunohistochemistry images were acquired using a Leica TCS SP8 confocal microscope equipped with an Andor iXon Ultra 888 monochromatic camera. The HC PL APO 40x/1.30 Oil CS2 (Leica Microsystems) objective was used for the acquisition. All figures were analyzed with Fiji and assembled in GIMP.

### Statistical analyses

All data are reported as mean ± SEM. Statistical analysis was performed using the GraphPad Software. Data groups from RT-qPCR experiments and those from measurements of outer limiting membrane (OLM)-Impg2 distance and OLM-ROS distance were compared by one-way ANOVA followed by Tukey’s test for multiple comparisons. The statistical significance level was set at p < 0.05. Values levels of statistical significance are described by asterisks (*p < 0.05; **p < 0.01; ***p < 0.001, ****p < 0.0001).

## Results

### IMPG2 sequence conservation analysis among vertebrates

Human IMPG2 is a 1241 residues protein with four topologically distinct regions: a 22 aa long signal peptide, an extracellular topological domain (residues 23 to 1099), a helical transmembrane domain (1100 to 1120), and a cytoplasmic topological domain (1121 to 1241). The extracellular region contains two SEA domains and two EGF-like tandem repeats, together with five hyaluronan-binding motifs. The protein is also a target for glycosylation and phosphorylation at different sites (UniProt database, www.uniprot.org/uniprot/Q9BZV3).

**Fig. 1:**
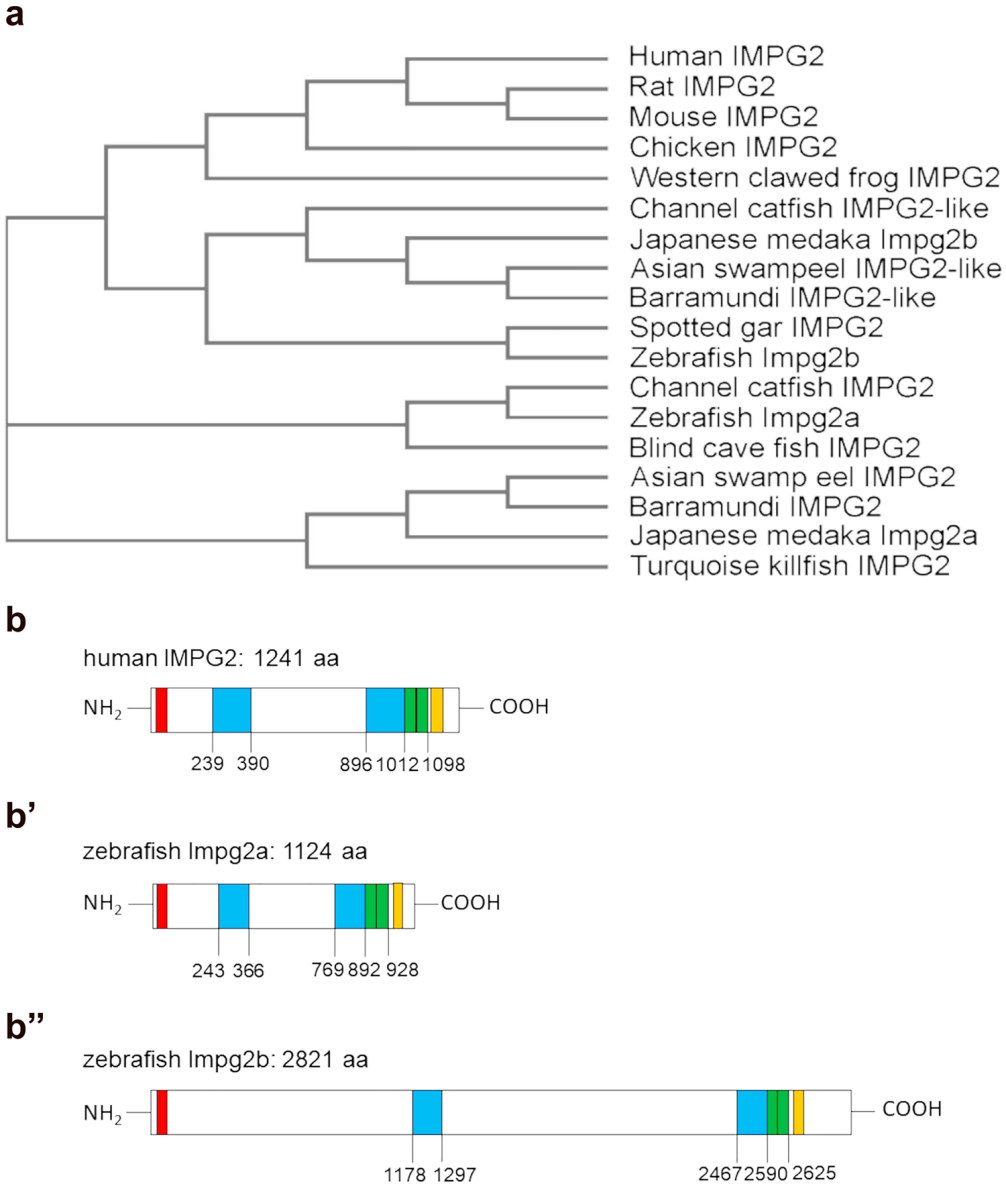
IMPG2 protein sequence conservation among vertebrates

We first investigated IMPG2 conservation during evolution by performing a protein–protein alignment (https://blast.ncbi.nlm.nih.gov/Blast.cgi) using as query the human IMPG2 sequence. We found statistically significant similarities only among jawed vertebrates (data not shown). We then proceeded to generate a phylogenetic tree by aligning the protein sequences of chosen vertebrate species, retrieving the different IMPG2 protein sequences from the NCBI database. We selected some of the most common species of various vertebrate groups to include in our analysis. We then performed a multiple sequence alignment and generated a phylogenetic tree (Fig. 1a), which reflects the distance in terms of protein sequence between the different vertebrate species. The length of the branches is directly correlated with the difference between the sequences. Most vertebrates, including humans, have only one *impg2* gene, while most extant bony fish have two, which cluster separately. Interestingly some teleosts, such as the Mexican blind cavefish (*Astyanax mexicanus*), have only the Impg2a paralogue, possibly due to the loss of the other paralogue after the whole-genome duplication event. In contrast, the spotted gar (which does not have any genome duplication) only has an Impg2b-like protein.

To understand in more detail the sequence conservation between the two zebrafish proteins and human IMPG2, we used the UniProt database. This analysis highlighted that the main domains of the human protein (SEA, EGF-like, and transmembrane) are conserved in both zebrafish paralogues (Fig. 1b, b’, b’’). Using the BLAST Alignment Tool, we found that both fish proteins share 65% identity with the region of the human protein where the conserved domains are located (residues 879–1238). These conserved domains were then analyzed in more detail by homology modelling.

#### SEA and EGF-like domains modelling in human IMPG2 and zebrafish Impg2a and Impg2b

**Fig. 2:**
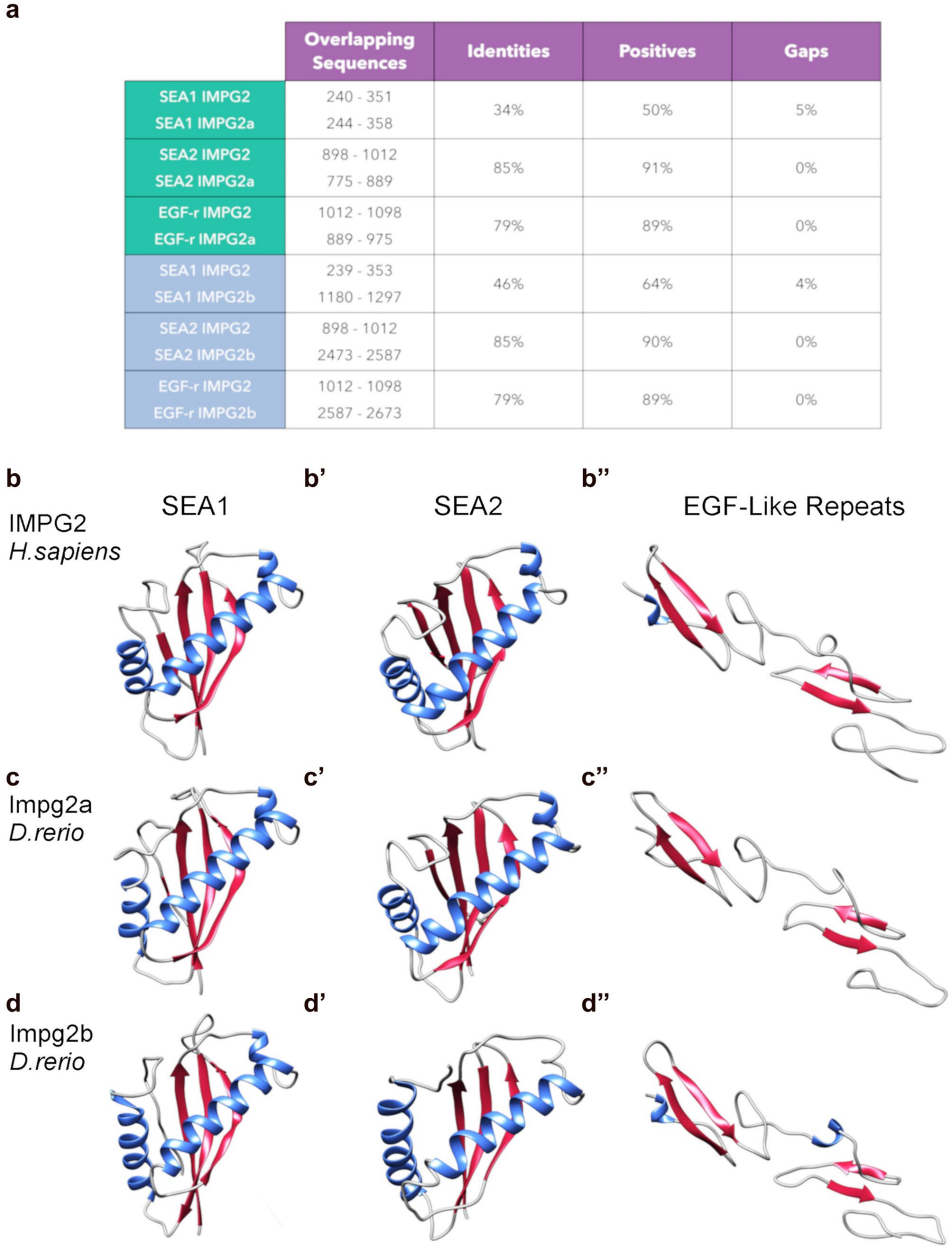
IMPG2, Impg2a and Impg2b conserved domains

Since little is known about the structure of the IMPG2 protein, we used iterative threading assembly refinement (iTasser) modelling to investigate the putative conformations of SEA and EGF-like conserved domains from their amino acid sequences. The following sequences of the human protein were submitted to the iTasser webserver: 239–390 (SEA1), 896–1012 (SEA2), and 1012–1098 (EGF-like tandem repeat). Domain identifications were obtained by checking Pfam (El-Gebali et al. 2019) and Prosite (Gasteiger et al. 2003) annotations. Then we used BLAST alignment to identify the sequences corresponding to the human domains in zebrafish Impg2a and Impg2b. The results are reported in Fig. 2. We also submitted these sequences (adding the non-overlapping residues at the terminals) to the iTasser web server for modelling. The best model proposed by iTasser for each domain was selected in terms of C-score. Figure 2b–d’’ shows the predicted structures. These analyses revealed a high (> 75%) sequence identity between the human IMPG2 and zebrafish Impg2a and Impg2b for the SEA2 and EGF-like domains (Fig. 2a). Conversely, the sequence identity between the human and zebrafish SEA1 domains display a much lower value (< 50%, Fig. 2a). Regardless of sequence identity, our modelling highlights a high structural similarity between the SEA and EGF-like domains of the human IMPG2 and the corresponding sequences of the zebrafish homologues Impg2a and Impg2b.

### *impg2a* and *impg2b* expression during development and in the adult

**Fig. 3:**
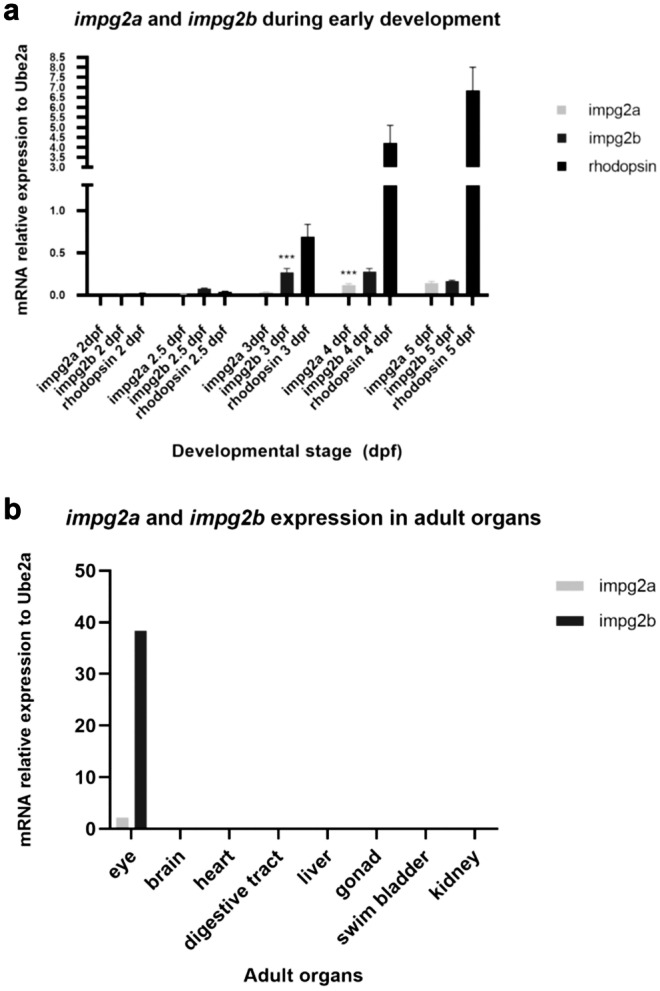
*impg2a* and *impg2b* expression during development and in the adult fish

We initially investigated *impg2a* and *impg2b* mRNA expression during early embryonic development and in the adult. RT-qPCR experiments were performed on RNAs extracted from pools of whole embryos at different developmental stages and from various dissected organs (eye, brain, heart, digestive tract, liver, gonads, swim bladder, kidney) of adult fish. Results reveal very low expression of *impg2b* at 2.5 days post fertilization (dpf) whilst *impg2a* mRNA is barely detected (Fig. 3a). Significant increase in *impg2a* and *impg2b* mRNAs can be observed at 3 dpf and 4 dpf, respectively (p < 0.001, Tukey’s test following one-way ANOVA) (Fig. 3a). This temporal pattern of expression of both *impg2a* and *impg2b* closely correlates with that of rhodopsin, a strongly expressed photoreceptor-specific gene starting to be expressed at 3dpf (Fig. 3a). Rhodopsin is synthesized in the photoreceptor inner segment (IS) and subsequently transported to the outer segment (OS), where it plays a central role in the phototransduction cascade (Huber and Sakmar 2008; Zhou et al. 2012). In the adult, *impg2a* and *impg2b* are specifically expressed in the eye whilst they could not be detected in any other organ we analyzed (Fig. 3b).

**Fig. 4:**
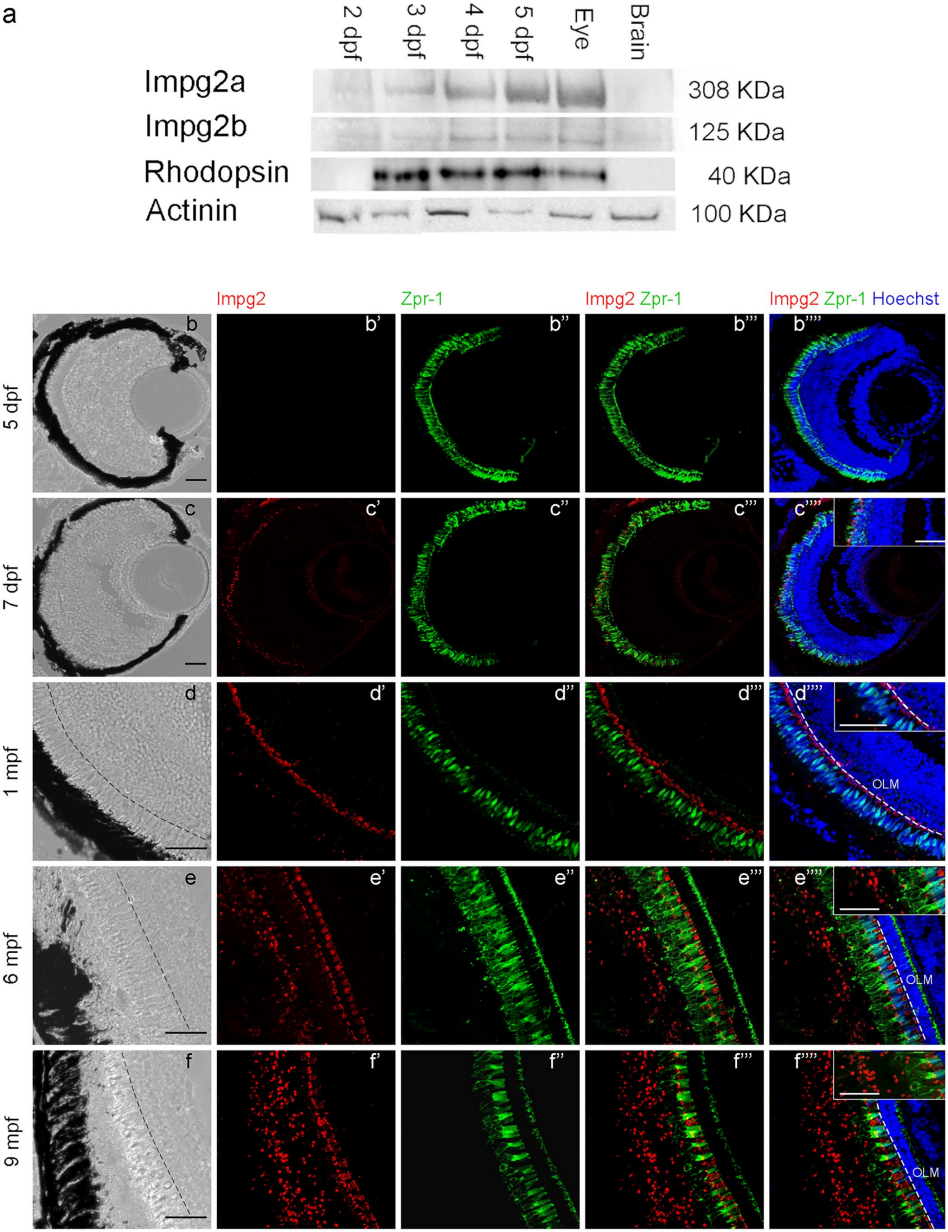
Impg2 localization during development and in adult fish

To study protein expression and localization, we took advantage of a human anti-IMPG2 antibody that recognizes an epitope conserved in both Impg2a and Impg2b zebrafish proteins. The immunogenic sequence in the human IMPG2 protein is localized within the SEA-2 domain (position aa. 900–950). As described in the above paragraph, this domain is conserved in both zebrafish paralogues, and thus is the epitope. Besides epitope sequence analysis, validation for usage in zebrafish was also performed by antibody dilution series, and incorporation of adequate negative controls in all experiments. We performed western blot experiments on pools of embryos at different developmental stages and pools of dissected brains and eyes of adult zebrafish. The expression of the two proteins becomes detectable at 3 dpf, when rhodopsin also starts being expressed (Fig. 4a). This observation further suggests that Impg2 expression accompanies photoreceptor maturation. Moreover, both Impg2a and 2b show a retina-specific expression, as observed for their mRNAs localization.

**Fig. 5:**
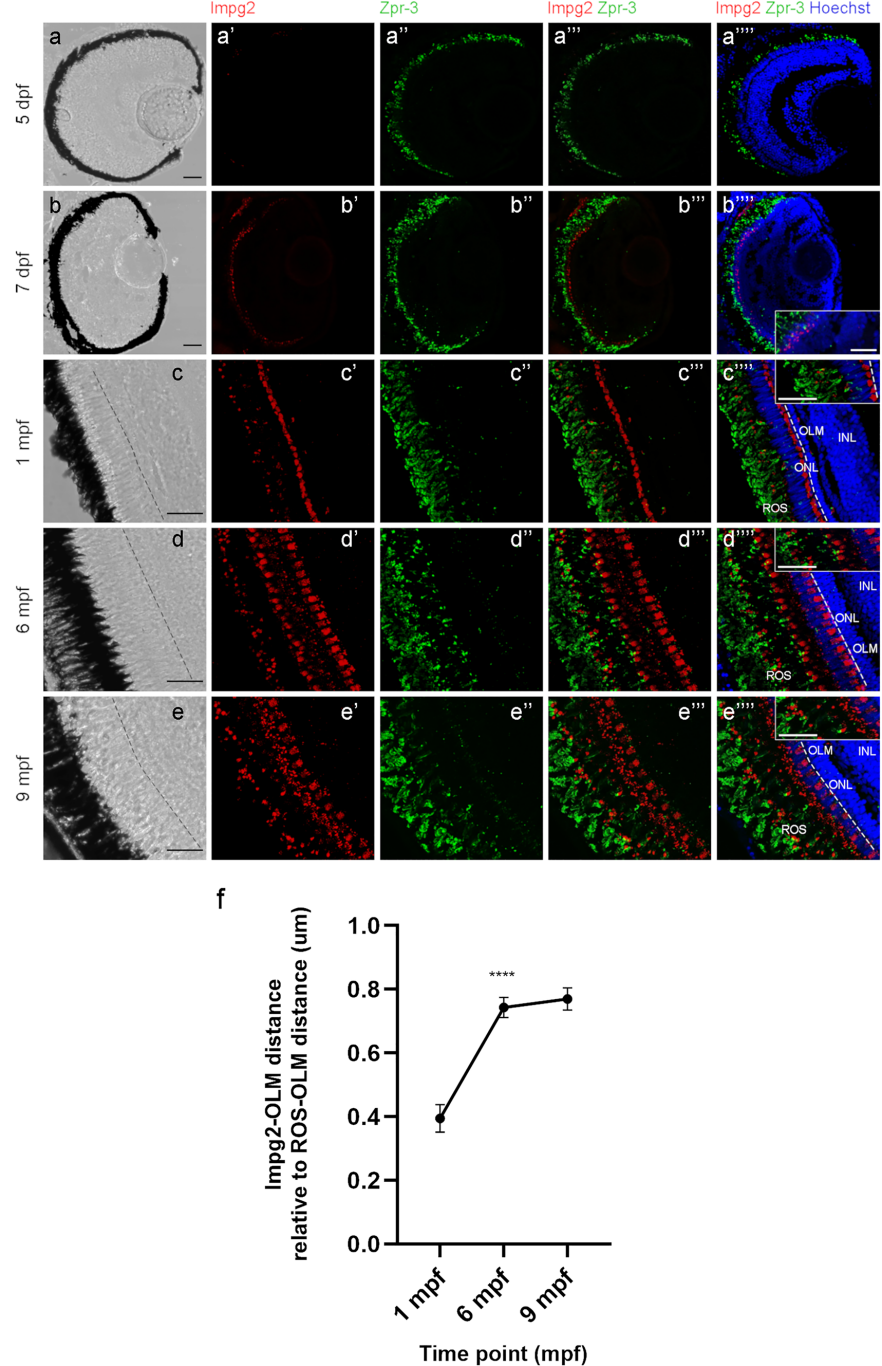
Length of Impg2 signal relative to OLM-ROS distance at different adult stages

To study their localization within the retina, we performed immunohistochemistry (IHC) experiments on sections of WT embryos at different developmental stages and adult dissected eyes, using the same anti-IMPG2 antibody used in western blots, which recognizes both zebrafish proteins. Thus, we will refer to Impg2 localization in general (Figs. 4b-f’’’’, 5a-e’’’’). To label photoreceptors, we used an anti-Zpr-1 antibody (Fig. 4b-f’’’’) that localizes to red/green double cones (DC) (Ali et al. 2020) and an anti-Zpr-3 antibody (Fig. 5a-e’’’’) localizing instead to rods and short double cones outer segments (OS) (Yin et al. 2012). Figure 4b-f’’’’ show that the Impg2 signal starts to be detected by the anti-IMPG2 antibody at 7 dpf (Fig c’,c’’’’) at the level of the outer nuclear layer (ONL), where photoreceptor cell bodies are located. At this stage, an overlay between Zpr-1 and Impg2 signal can be detected (Fig. 4c’’’,c’’’’). The Impg2 signal becomes more robust at 1 month post-fertilization (mpf) and is mainly detected in a domain consistent with the positioning of the photoreceptor’s cell bodies located externally to the outer limiting membrane (OLM, Fig. 4d’-d’’’’). This structure subdivides the ONL into a more internal sublayer containing rod and short single cone (SSC) nuclei and a more external sublayer containing long single cone (LSC) and double cone nuclei (Lagman et al. 2015; Angueyra and Kindt 2018). As photoreceptors undergo maturation and elongation, the Impg2 signal located in the photoreceptors cell bodies region apically to the OLM (Fig. 4e’-e’’’’, f’-f’’’’, 6 and 9 mpf) remains present. Concomitantly, a dotted Impg2 signal becomes prominent at variable distances in the more external region toward photoreceptors outer segments (Fig. 4e’-e’’’’, 4f’-f’’’’, 6 and 9 mpf).

To understand the localization of Impg2 signal in more detail, we compared it to that of Zpr-3, which labels outer segments (OS) of both rods and short double cones (Fig. 5a-e’’’’). Results show that at 1 mpf the weak dotted Impg2 signal localizes to the OS (Fig. 5c’-c’’’’), while the stronger Impg2 signal is localized to the basal domain consistent with its positioning at the level of the presumptive photoreceptor cell bodies, confirming the previous analysis. At 6 and 9 mpf most of the signal is detected in the OS region, and this Impg2 signal increases with age (Fig. 5d’-d’’’’, e’-e’’’’, 6 mpf and 9 mpf).

The dynamic distribution of the dotted Impg2 signal observed in retinas of different ages might be due to an effective rearrangement in its localization or to its adjustment to photoreceptor’s elongation during retinal growth and maturation. To discriminate between these possibilities, we measured the distance between the OLM and the outer limit of the Impg2 staining and compared it to the distance between the OLM and the outer limit of ROS identified by the external boundary of Zpr-3 staining. Results (Fig. 5f) suggest a significant displacement of Impg2 signal occurring between 1 and 6 mpf (p < 0.0001, one-way ANOVA). No significant increase is observed between 6 and 9 mpf, in spite of photoreceptor elongation (Fig. 5f), indicating that Impg2 becomes progressively displaced towards the outer photoreceptor segment between 1 and 6 mpf.

## Discussion

Extracellular matrix plays a key role in retinal function and disease (Nichol et al. 1995; Al-Ubaidi et al. 2013; Reinhard et al. 2015, 2017). However, little is known about the function and the structure of many of its components, such as the proteoglycan IMPG2. This study begins to fill this gap with the characterization of the human IMPG2 protein structure in comparison with its zebrafish counterpart. Zebrafish is an increasingly popular model organism for the study of human retinal degenerative disorders since, unlike mouse, it has a cone-dominant vision with a retinal structure similar to the human macula (Gestri et al. 2012; Chhetri et al. 2014). By comparing human IMPG2 with its two zebrafish paralogues, Impg2a and Impg2b, we highlighted the presence of SEA and EGF-like conserved domains in evolutionarily distant vertebrate species such as Homo sapiens and Danio rerio.

IMPG2 is a vertebrate-specific gene, as it is not present in other animal groups. Among vertebrates, teleosts are the only group showing two Impg2 paralogues, consistently with the whole-genome duplication that occurred in the common teleosts ancestor (Glasauer and Neuhauss 2014). In fact, the spotted gar (*Lepisosteus oculatus*), which diverged from teleost ancestors before the genome duplication, has only one *impg2* gene. Moreover, not all the teleosts that we analyzed have two paralogues. For example, the killifish (*Notobranchius furzeri*) and the blind cavefish (*Astyanax mexicanus*) have only one *impg2* gene. This is consistent with the idea that, after the genome wide duplication, the two different paralogues can have different fates in different species, and that the most likely outcome is the loss of functionalization of one of the two (Glasauer and Neuhauss 2014). The function – or the functions – of the two zebrafish *Impg2* paralogues *impg2a* and *impg2b* will need to be elucidated in future analyses.

Homology modelling of SEA and EGF-like conserved domains in human IMPG2 and zebrafish Impg2a and Impg2b shows structure similarity of the domains in the two species. With this level of structural prediction confidence, no substantial differences between the domains of the different species are evident, or rather, nothing that could not be ascribed to the uncertainty of the iTasser prediction. So, this homology modelling study is a confirmation of the expected structure of the specific portions of the proteins, and therefore also of the function of those regions. The SEA domain is an extracellular region found in secreted or transmembrane proteins. No clear function has been established, but it is associated with sites of extensive O-glycosylation and might regulate carbohydrate binding (Bork and Patthy 1995). The EGF-like extracellular domains have been implicated in different cellular processes such as growth, differentiation and apoptosis (Engel 1989; Park et al. 2008). Their presence suggests for Impg2 a potential role in protein–protein interactions and sugar binding that might contribute to regulate IPM stability and function (Chen et al. 2003).

Finally, we report for the first time a dynamic pattern of zebrafish Impg2a and Impg2b expression and localization during development and in adulthood. Our results show expression of impg2a and impg2b mRNAs and proteins starting from 3 dpf, and an expression in the adult that is restricted to the eye. Published transcriptomic data report expression of both genes also in the pineal gland (https://snengs.nichd.nih.gov/) (Chang et al. 2020). This is not in contrast with our data, as pineal-specific transcripts might be too diluted to be observed in our experiments (in embryos), or the pineal gland itself might have been lost during brain dissection (in the adult).

As shown by western blots, the antibody we used, although raised against the human protein, is able to recognize in zebrafish two proteins of the appropriate size for Impg2a and 2b. It was thus possible to study their localization by IHC. However, since the epitope against which the antibody was raised is conserved in both zebrafish proteins, we were not able to discriminate between Impg2a and 2b expression. Therefore, we will refer to Impg2 expression in general, without distinguishing between the two proteins. Our data show that zebrafish Impg2 proteins are found exclusively in the photoreceptor layer. Interestingly, their expression pattern changes with age. Indeed, the signal accumulates at the level of the photoreceptor cell bodies during larval development, with only a limited expression in the outer segment region. This latter signal becomes predominant between 1 and 6 mpf, expanding along the outer segments of both rods and cones, as shown by the comparison with Zpr-1 and Zpr-3 expression. Impg2 signal does not overlap with either of the other two proteins, suggesting a different cellular/extracellular localization. In mouse (and humans), IMPG2 is considered to be a transmembrane or extracellular protein (Salido and Ramamurthy 2020; Chen et al. 2004). Specifically, a recent study demonstrated that the SEA-2 domain in the mouse IMPG2 protein has autoproteolytic capacity, giving rise to a secreted peptide and to a small membrane-bound peptide, and that this proteolysis is crucial in the maturation of IMPG2 (Mitchell et al. 2022). Our data could indicate a secretion of the protein that increases with age. These data are consistent with the results of previous studies regarding the localization of IMPG2 in the rodent retina (Salido and Ramamurthy 2020; Xu et al. 2020).

IPM structural integrity is fundamental for its function. According to the data presented in this work and the published literature regarding Impg2 in other species (Salido and Ramamurthy 2020; Xu et al. 2020), we can speculate that it plays a role in the trafficking between photoreceptors and RPE, and that an alteration of its function could lead to IPM disruption, followed by RPE dysfunction. However, our analysis is the first demonstrating a dynamic localization of the Impg2 protein over time, thus raising interesting questions on the possibility that it might play different roles during the development and maturation of the retina.

This work combines structural analysis of the conserved domains of human IMPG2 and zebrafish Impg2a and Impg2b, and expression analysis of *impg2a* and *impg2b* mRNAs and proteins in zebrafish embryos and adults, providing insights into the biology of these disease-related genes. Our data allow a better understanding of the physiology of the IPM and the interactions among its components, laying the basis for further studies on the molecular mechanisms of retinal diseases, in turn leading to innovative therapeutic approaches.

## Data Availability

All data generated or analysed during this study are included in this published article (and its supplementary information files).

## List of abbreviations

arRP: Autosomal recessive Retinitis pigmentosa
dpf: Days-post-fertilization
EGF: Epidermal growth factor
ER: Endoplasmic reticulum
IHC: Immunohistochemistry
IPM: Interphotoreceptor matrix
IMPG1: Interphotoreceptor proteoglycan 1
IMPG2: Interphotoreceptor matrix proteoglycan 2
IRBP: Interphotoreceptor retinoid-binding protein
IRD: Inherited retinal dystrophy
IS: Inner segment
mpf: Months-post-fertilization
OLM: Outer limiting membrane
ONL: Outer nuclear layer
OS: Outer segment
SEA: Sperm protein, Enterokinase and Agrin
VMD: Vitelliform macular dystrophy
